# Yield and Quality of Walnuts Subjected to Deficit Irrigation in Mountainous Water-Starved Environments

**DOI:** 10.3390/plants14121777

**Published:** 2025-06-10

**Authors:** Víctor Hugo Durán Zuazo, Belén Cárceles Rodríguez, Esther Sendra, Ángel Antonio Carbonell-Barrachina, Leontina Lipan, Francisca Hernández, Baltasar Gálvez Ruiz, Iván Francisco García-Tejero

**Affiliations:** 1 IFAPA Centro “Camino de Purchil”, Camino de Purchil s/n, 18004 Granada, Granada, Spain; victorh.duran@juntadeandalucia.es (V.H.D.Z.); baltasar.galvez@juntadeandalucia.es (B.G.R.); 2 Research Group “Food Quality and Safety”, Centro de Investigación e Innovación Agroalimentaria y Agroambiental (CIAGRO-UMH), Universidad Miguel Hernández, Carretera de Beniel, km 3.2, 03312 Orihuela, Alicante, Spain; esther.sendra@umh.es (E.S.); angel.carbonell@umh.es (Á.A.C.-B.); leontina.lipan@goumh.umh.es (L.L.); francisca.hernandez@umh.es (F.H.); 3 IFAPA Centro “Las Torres”, Carretera Sevilla-Alcalá del Río km 12,2, 41200 Alcalá del Río, Sevilla, Spain; ivanf.garcia@juntadeandalucia.es

**Keywords:** deficit irrigation, walnut quality, fatty acid profile, Mediterranean semiarid areas, mountainous farming

## Abstract

Walnut (*Juglans regia* L.) exhibits a high sensitivity to water deficit, making it crucial to comprehend this characteristic in order to optimize irrigation strategies to improve its productivity. Deficit irrigation is widely used under drought conditions to achieve water savings goals. This study examines the impact of sustained deficit irrigation (SDI) strategies—applying 33%, 50%, or 75% of the crop water demand—on yield and quality parameters of two walnut cultivars (Chandler and Cisco) over a three-year monitoring period. These treatments were compared against control trees receiving full irrigation at 100% of crop water requirements (C_100_). The nut yield was significantly and proportionally reduced under the SDI treatments. In the experiment, the average yield for cv. Chandler amounted to 6.7, 6.4, and 12.2 kg tree^−1^ under SDI_33_, SDI_50_, and SDI_75,_ respectively, which were less than 13.9 kg tree^−1^ in the C_100_ plot. Similarly, cv. Cisco yielded 8.0, 11.6, 11, and 15.6 kg tree^−1^ under SDI_33_, SDI_50_, SDI_75_, and C_100_, respectively. These findings indicate that the cultivar Cisco exhibits greater tolerance to moderate and intermediate levels of water deficit. Furthermore, the SDI treatments notably influenced several morphological and physicochemical kernel parameters. The key affected attributes include the weight, size, color, profiles of specific sugars, and mineral content (notably potassium, iron, and zinc), as well as the composition of unsaturated fatty acids (palmitoleic and cis-vaccenic) and polyunsaturated fatty acids (linoleic and α-linolenic), with pronounced effects observed particularly under the SDI_75_ treatment. Thus, deficit irrigation did not drastically affect the kernel quality parameters, and it is also possible to augment them by selecting the appropriate water stress level. Therefore, for both walnut cultivars, approximately 25% of the irrigation water (SDI_75_), equivalent to an average of 1681 m^3^ ha^−1^, can be conserved relative to the total crop water requirement without negatively impacting walnut tree performance in the short to medium term. Here, we show the key role of adjusting irrigation practices by stressing the benefits of SDI that can save water, foster water productivity, and boost walnut health-promoting phytochemicals.

## 1. Introduction

The walnut (*Juglans regia* L.) tree is extensively cultivated in temperate regions worldwide and is one of the most marketable and important edible nuts [1,2,3]. This woody fruit tree belongs to the family Juglandaceae that originated in Persia [4,5]. In 2021, China had the highest area harvested of 279,853 ha, followed by the United States of America (157,829 ha), Turkey (153,520 ha), Mexico (111,589 ha), Burkina Faso (91,370 ha), and Iran (53,504 ha) [6]. In Spain, the area devoted to walnut cultivation amounted to 15,285 ha, with ~53% of this total area being irrigated [7]. In this context, irrigation is one of the most critical agronomic practices and plays a pivotal role in the successful cultivation of walnut trees [8,9,10,11]. Historically, and under varying climatic conditions, a significant proportion of walnut plantations were established without irrigation. However, given the walnut tree’s positive response to water availability, manifested in both enhanced vegetative growth and substantial increases in nut yield, most commercial walnut orchards today are irrigated. Walnuts are widely regarded as a healthy food choice due to their bioactive composition and medicinal properties. Nutritionally, they are a rich source of fatty acids, polyphenols, flavonoids, tocopherols, essential amino acids, and minerals [12,13,14,15]. Notably, virgin walnut oil is characterized by a high content of monounsaturated (MUFAs) and polyunsaturated fatty acids (PUFAs) [16,17]. According to Das [18], PUFAs, particularly linoleic acid and α-linolenic acid, constitute more than half of the total lipid content in walnuts. This is particularly significant, as the human body cannot synthesize these essential fatty acids. Walnut is rich in tocopherols and essential fatty acids such as omega-3 (Ω3) and omega-6 (Ω6), with important effects on human health [19,20]. Among the phytochemicals, the polyphenol, flavonoid, and tocopherol contents in walnuts possess numerous health-promoting attributes, such as their ability to act as antioxidants and provide antibacterial, antifungal, anti-inflammatory, anti-aging, anticancer, and neuroprotective benefits [21,22].

Variations in phytochemical composition among different walnut cultivars have been documented for the major commercially cultivated varieties [23,24]. The component variability among cultivars in different locations and climate zones is especially apparent [25]. In this regard, numerous studies have identified significant differences in the levels of fatty acids, minerals, and polyphenols among walnut cultivars [21,26,27]. In addition to cultivar-specific effects, climatic conditions have been shown to directly influence the phytochemical composition. Research by Rabadán et al. [23,28] demonstrated that seasonal climatic variability had a greater impact on the concentrations of fatty acids, polyphenols, tocopherols, and essential minerals (e.g., iron) than the differences observed between cultivars. According to Fuentealba et al. [29], lower minimum temperatures caused an increase in the synthesis of unsaturated fatty acids, while rainfall directly impacted the contents of phenolic compounds during kernel-growing seasons [30]. The year and genotype variability also led to alterations in the tocopherol content of walnuts, as stated by Kodad et al. [31].

Today, certain walnut growing regions could be impacted by a changing climate, particularly the European regions in the Mediterranean and temperate continental climates [32,33]. In this context, the effects of climate change are expected to manifest most prominently through changes in water availability for agriculture, with potentially significant and uneven consequences for farming activities [34,35]. Specifically, in the Mediterranean basin, Gorguner and Kavvas [36] project a substantial decline in water availability in the coming decades. This trend, combined with the ongoing expansion and intensification of agricultural land use, is anticipated to lead to an increased demand for water resources [37,38]. Arid and semiarid regions are currently experiencing water scarcity for agricultural purposes, which represents the most critical environmental constraint for woody fruit crops, adversely impacting both productivity and sustainable development [39,40]. Therefore, water availability and scarcity clearly highlight the necessity for irrigated crops in Mediterranean regions to adopt alternative techniques in order to enhance resilience under conditions of water shortage [41]. The walnut is a tree with a high water demand and is markedly susceptible to water stress [42,43,44]. Accordingly, water stress negatively affects its growth and productivity [45,46]; therefore, this may reduce the viability of current mature orchards and the establishment of new ones.

Under drought conditions or high leaf-to-air vapor pressure deficit conditions, walnuts have good stomatal regulation [47]; preventing the stem water potential from dropping below −1.4 MPa is crucial, as this threshold corresponds to the onset of xylem cavitation, as reported by Tyree et al. [48] and Cochard et al. [49]. Due to this behavior, the regulated deficit irrigation (RDI), which withholds water at specific growth stages, has not proven to be as effective in walnut as in other woody crops such as almond, pistachio, vineyard, or olive [50]. For these crops, properly timed and controlled levels of water deficit decreased the amount of irrigation water and benefited the yield or crop quality. By contrast, sustained deficit irrigation (SDI) refers to a water restriction strategy in which the crop receives a lower and uniform amount of irrigation water. That is, the water is applied below the crop evapotranspiration, developing a progressive stress in the plant throughout the season; furthermore, SDI is the simplest strategy used by farmers in areas with reduced available water resources. Consequently, studies on the responses and adaptations of walnut trees to water stress induced by deficit irrigation, as well as their implications for yield and quality, are essential. Conversely, it is well-established that a water deficit can enhance the accumulation of secondary metabolites in plants, thereby improving the functional properties of edible products [51,52]. These secondary metabolites are key contributors to the antioxidant activity of walnuts, playing crucial roles in plant adaptation to environmental stresses and resistance to biotic factors while also offering significant benefits for human nutrition and health [53]. Specifically, under a Mediterranean climate characterized by hot and dry summers and predominantly humid winters, irrigation plays a critical role in compensating for the water deficits caused by high crop evapotranspiration. In future scenarios, the increasing demand for water resources will necessitate the development of strategies to optimize water productivity (WP) and maximize water savings while sustaining crop yield. In this context, deficit irrigation, applying controlled water restrictions that minimally affect crop yield without compromising sustainability, has emerged as an effective approach to enhance water productivity (WP) [40]. Recent studies conducted across diverse environments have demonstrated the benefits of deficit irrigation in improving the WP and fruit quality in various nut tree species, including almond [54], hazelnut [55], cashew [56], macadamia [57], pecan [58], pistachio [59], and pine nut [60].

This study evaluated the effects of SDI on the yield and quality of two walnut cultivars (Chandler and Cisco) under water stress conditions over three consecutive growing seasons in an orchard situated in the semiarid mountainous region of southeastern Spain.

## 2. Results and Discussion

### 2.1. Walnut Yield Response to Deficit Irrigation

**Figure 1:**
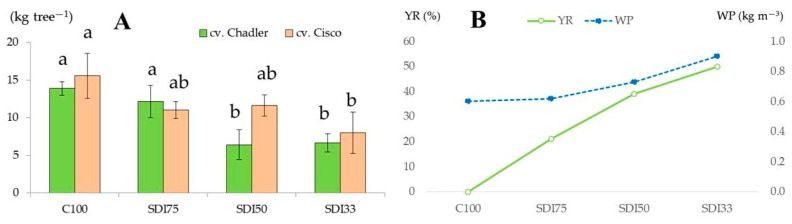
Response of walnut yield to deficit irrigation treatments during a three-year monitoring period: (**A**) average yield reduction (YR) and (**B**) water productivity (WP) for both walnut cultivars. SDI, sustained deficit irrigation; SDI_33_, at 33% ET_C_; SDI_50_, at 50% ET_C_; SDI_75_, at 75% ET_C_; and C_100_, control at 100% ET_C_. Values associated with the same letter among columns are not significantly different using Tukey’s least significant difference test (*p* < 0.05). Vertical bars represent the standard deviation.

Over the three monitoring seasons, the average yields of cv. Chandler walnuts from water-stressed trees under the SDI_33_, SDI_50_, and SDI_75_ treatments were 6.7, 6.4, and 12.2 kg tree^−1^, respectively. The yields under SDI_33_ and SDI_50_ were significantly lower compared to the non-stressed control trees (C_100_), which produced an average of 13.9 kg tree^−1^ (Figure 1A). Similarly, for cv. Cisco, the walnut yields for SDI_33_, SDI_50_, SDI_75_, and C_100_ were 8.0, 11.6, 11.0, and 15.6 kg tree^−1^, respectively. Potentially, in terms of productivity per area in such mountainous walnut plantations, the SDI_33_, SDI_50_, SDI_75_, and C_100_ for cv. Chandler can be 1.8, 1.7, 3.3, and 3.8, and for cv. Cisco, they can be 2.2, 3.2, 3.0, and 4.2 t ha^−1^, respectively. This suggests that cv. Cisco seems to be more tolerant to water stress than cv. Chandler, even when the reduction in irrigation water is 50%. In this sense, Aletà et al. [61] reported yields for cv. Chandler and non-water-stressed trees in Northern Spain between 2.5 and 1.85 t ha^−1^. Due to the alternate bearing nature of walnut trees, the impact of water stress induced by deficit irrigation is more accurately assessed using yield data averaged across the monitored seasons. Accordingly, when averaged over time, the yield reductions for both walnut cultivars under moderate (SDI_75_), medium (SDI_50_), and severe (SDI_33_) water stress conditions were 21, 39, and 50%, respectively, relative to the non-stressed control treatment (C_100_) (Figure 1B). These findings suggest that the yield of the non-stressed trees was slightly less affected by the alternate bearing pattern, whereas the yield of the water-stressed trees experienced a more pronounced decline during off-bearing seasons.

Along these lines, according to a two-year monitoring study of Kornov et al. [62], the yield reductions due to irrigation regimes based on 150% ET_C_, 50% ET_C_, and non-irrigated trees were 24.4, 38.6, and 59.7%, respectively, compared to the full-irrigated walnut trees at 100% ET_C_. Thus, the decline in yield was important and corroborated the sensitivity of the walnut to water supply. Similarly, Goldhamer et al. [63] reported the yield for walnut irrigated with 100% ET_C_, 66% ET_C_, and 33% ET_C_ at 5.0, 3.3, and 2.5 t ha^−1^, respectively. A study by Calvo et al. [64] for two monitoring seasons revealed the impact of a water deficit using four applied water treatments of 50, 75, 100, and 125% of ET_C_, yielding 2.50–3.14, 2.97–3.50, 3.14–3.75, and 3.28–3.83 t ha^−1^, respectively. In addition, Goldhamer et al. [65], in a six-year study, found that the yield of Chico walnut was reduced by 20 to 40% (SDI strategy) when trees were supplied irrigation at 33% and 66% of ET_C_, respectively; two years of irrigation at full ET_C_ were required before the walnut trees returned to near-normal yield potential. The findings of the present experiment aligned with those of Perulli et al. [11], who reported that the adoption of deficit irrigation rates at 75 and 50% ETc did not affect Chandler walnut quality parameters and yield to a high degree. Moreover, applying both irrigation strategies improved water-use efficiency during a four-year monitoring period.

**Figure 2:**
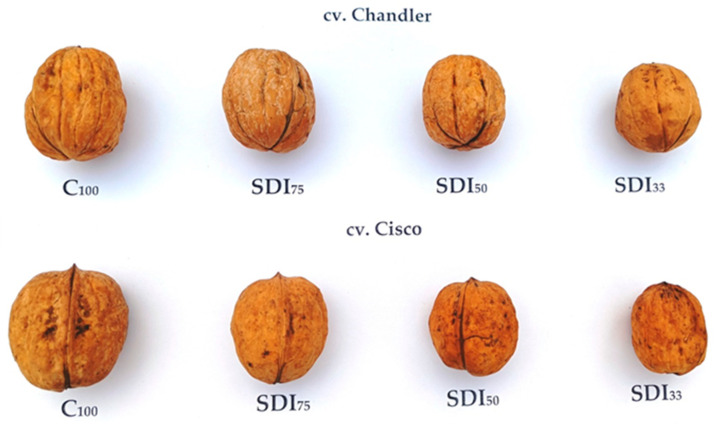
Walnut cultivars irrigated with different sustained deficit irrigation (SDI) levels. SDI, sustained deficit irrigation; SDI_33_, at 33% ET_C_; SDI_50_, at 50% ET_C_; SDI_75_, at 75% ET_C_; and C_100_, control at 100% ET_C_.

As expected, a reduction in yield and nut development occurred due to deficit irrigation; the visual impact of the latter on the nut size of the studied walnut cultivars with respect to the full-irrigated control nuts is shown in Figure 2. Under the conditions of this experiment, moderate water stress, corresponding to 75% of crop evapotranspiration (ET_C_), appears to be a viable strategy for reducing irrigation water use without substantially compromising the productivity of either walnut cultivar. However, the Cisco walnut was more tolerant to water stress at 50% ET_C_, yielding almost similar results to under 75% ET_C_. This observation aligns with the findings of Buchner et al. [66], who reported that Chandler walnut trees are not well suited for deficit irrigation due to their pronounced sensitivity to water stress.

### 2.2. Water Productivity

The average WP for cv. Chandler under SDI_33_, SDI_50_, SDI_75_, and C_100_ during the study period was 0.82, 0.52, 0.66, and 0.56, respectively, and for cv. Cisco, it was 0.98, 0.94, 0.59, and 0.63 kg m^−3^, respectively. Comparable improvements in water productivity through deficit irrigation, relative to fully irrigated trees, have been documented for walnut trees by Guiqing et al. [67] and Perulli et al. [11]. Cohen et al. [68] studied three irrigation strategies: control at 100% ET_C_, regulated deficit irrigation (RDI) with 20% ET_C_ (irrigated June–September), and SDI at 70% ET_C_ throughout the growing season. Both the RDI and SDI strategies reduced the walnut yield by 40% with respect to the control. This meant an increase in the WP for dry in-shell yield for the control, RDI, and SDI of 2.4, 5.6, and 4.7 kg ha^−1^ mm^−1^, respectively. Similarly, Calvo et al. [64] calculated the WP function for walnut dry in-shell yield and total applied water over a season, estimating a maximum yield of 9.93 t ha^−1^ with a maximum irrigation dose of 1350 mm ha^−1^. Finally, in a study by Abdullah et al. [10] on irrigated walnut orchards, they saved around 1700 m^3^ ha^−1^ of irrigation water, with average yield reductions of 145 kg ha^−1^ and a higher WP of roughly 0.02 kg m^−3^. The responses of walnut productivity to different irrigation water doses are highly variable and sometimes contradict logic; however, these are field experiments, in which there can be highly uncontrollable surrounding or environmental factors.

In relation to the water irrigation amount, Sadeghi-Majd et al. [69] reported drip irrigation water for walnuts at 7000 m^3^ ha^−1^; the best yield was produced when 100% of water needs were met, and increasing the irrigation by 25 or 30% unnecessarily enhanced the plant development and growth [68]. In addition, based on their findings, a 20% reduction in irrigation reduced the yield marginally but increased the crop quality, which coincides with the results of the present experiment with SDI_75_ (5042 vs. 6723 m^3^ ha^−1^ of control plot). A lower amount of water of 4130 m^3^ ha^−1^ for walnuts was reported by Xue et al. [70]. According to Zhang et al. [71], optimal walnut tree growth and development can only be achieved with drip and sprinkler irrigation and not with flood or rainfed systems. Similarly, Apáti et al. [72] stated that the effectiveness of the irrigation system in terms of walnut yield showed the following trend: sprinkler (2210 kg ha^−1^) > drip (2150 kg ha^−1^) > flood (1620 kg ha^−1^). In another study, Lampinen et al. [73] showed that the impact of pauper irrigation on yield was different and depended crucially on the tree’s age, cultivar, soil conditions, cultivation techniques, etc. Finally, Fulton [8], through a study on walnut trees in California (USA), reported that the walnut’s seasonal water need amounted to 10,620 m^3^ ha^−1^, which is much higher than that used for the full-irrigated trees in the present study.

In general terms, the implementation of sustained deficit irrigation at 75% of ET_C_ enhanced the WP and moderately controlled the vegetative growth without considerable negative implications on the walnut yield and with important water irrigation savings of about 1681 m^3^ ha^−1^.

### 2.3. Walnut Quality Parameters

#### 2.3.1. Morphological and Physical Features

Table 1 presents the effects of the irrigation regime and cultivar on the morphological and physical characteristics of the walnuts studied. The irrigation regime induced significant differences in walnut weight, kernel thickness, color, and kernel cutting force. In terms of the cultivar factor, significant differences were observed for all morphological parameters, except for the kernel weight, length, and thickness. The deficit irrigation treatments, particularly the SDI_33_, significantly reduced the weight and size of walnuts with respect to the C_100_ plot; similarly, important alterations for the a* and b* coordinates, hue, and cutting force were found. In general, the decline in the morphology parameter values was lower for walnuts at SDI_75_, and the pattern was in line with the level of water stress imposed (Figure 2). In addition, the water-stressed walnuts have higher L* coordinate values with respect to the non-stressed, and the lowest value for a* was denoted for the severely water-stressed SDI_33_ trees. A harder texture was determined for walnuts from SDI_75_ (33.7 N), and a softer texture was found for the remaining treatments, including the control. In general, cv. Cisco produced walnuts with the highest weight and size, and cv. Chandler had significantly lower values for all color parameters and higher hardness (31.1 N), in contrast to the number of fractures (36.9 vs. 34.2). According to Charrier et al. [74], walnut vegetative growth takes place fully in spring during the stage between bud break and endocarp hardening, defining the fruit numbers and size. In other words, the reduction in walnut quality, particularly in terms of size, can be attributed to potential water deficit conditions [75]. Pakrah et al. [76] provided evidence that water stress during the maturation phase may affect kernel color due to elevated temperatures caused by reduced hydraulic conductivity, leading to the chemical and enzymatic oxidation of phenolic compounds in the walnut kernel pellicle. Correspondingly, despite the decline in kernel color quality, the nutritional value of walnuts may improve significantly in warmer seasons or environments, such as semiarid Mediterranean regions, consistent with the findings of Calvo et al. [77].

Texture parameters are widely recognized as critical factors influencing consumer acceptance. In this context, both the deficit irrigation treatment and cultivar had significant effects. Overall, the SDI_75_ treatment resulted in notable increases in kernel hardness. Among the cultivars, Chandler exhibited the most favorable texture characteristics, demonstrating the highest hardness values. The analysis of color coordinates indicated that cv. Cisco possessed a lighter skin tone, while cv. Chandler exhibited a darker coloration. Specifically, cv. Cisco showed the highest a* values (indicating a more reddish hue) but the lowest b* values (indicating less yellow or more bluish tones). The interaction between the irrigation regime and cultivar resulted in significant differences across all the measured parameters. Notably, substantial variations in key morphological traits were observed for both cultivars, particularly under severe water stress conditions (SDI_33_), consistent with the levels of water deficit imposed. However, compared to the control trees (C_100_), no significant differences were detected for walnuts subjected to SDI_50_ in cv. Cisco, and a similar trend was observed for SDI_75_ in cv. Chandler. These results are consistent with the findings reported for other nut species, including hazelnuts [55], pistachios [59], almonds [54], macadamias [78], and walnuts [66], where many quality attributes remained largely unaffected by moderate or medium deficit irrigation treatments. Conversely, while irrigation treatments significantly influenced both the kernel ratio and dry weight, the cultivar factor alone did not exert a notable effect, although their interaction proved statistically significant (Table 2). Within the deficit irrigation regimes, SDI_75_ achieved the highest kernel ratio at 40.7%, whereas SDI_50_ resulted in the highest dry weight, measuring 5.49% among SDI strategies. These outcomes may be attributed to a decreased fruit load in trees subjected to water stress, potentially facilitating a more efficient kernel-filling process due to the reduced number of fruits per tree [79]. In terms of cultivar variation, no statistically significant differences were observed for either parameter.

The water activity (a_w_) was significantly influenced by the irrigation treatments, while the cultivar factor had no notable effect (Table 2). All deficit irrigation regimes led to a reduction in a_w_ compared to the fully irrigated control, with the most pronounced decrease observed under SDI_33_. Despite these reductions, all measured a_w_ values remained within the optimal range for the safe storage of tree nuts under cool and dry conditions [80]. Water activity plays a critical role in lipid oxidation in walnuts; maintaining lower a_w_ levels helps to minimize oxidative rancidity during storage [81]. In this regard, Boaghi et al. [82] reported that the highest lipid oxidation occurred in walnuts stored at a_w_ ranges of 0.00–0.28 and 0.48–1.00. Thus, deficit irrigation appears to promote lower a_w_ levels, potentially enhancing the postharvest stability and storage quality of the nuts. 

#### 2.3.2. Antioxidant Activity and Total Phenolic Content

Table 3 presents the antioxidant activity (AA) and total phenolic content (TPC) observed under various deficit irrigation strategies throughout the monitoring period. The applied irrigation treatments and walnut cultivars exhibited highly significant effects (*p* < 0.001) on both parameters.

The AA was determined through three assays: ABTS^•+^, DPPH^•^, and FRAP. The ABTS^•+^ results showed significant differences among cultivars and intermediate irrigation treatments, with no significant changes for SDI_33_ in relation to the control walnuts. The DPPH^•^ activity was reduced by the severe water-stressed treatments, but no significant impact was observed for the remaining treatments. In contrast, in the FRAP assay, SDI_33_ maintained the same level as the control. Regarding the cultivar factor, Cisco showed the highest values for ABTS^•+^, DPPH^•^, and FRAP, demonstrating the importance of cultivar identity. This result is in agreement with Bolling [83], who reported that different walnut cultivars have their own antioxidant characteristics.

Overall, in terms of irrigation strategies, one can retain the same value as the non-stressed walnuts for DPPH^•^, as well as for FRAP under SDI_75_ and SDI_50_; however, these same strategies decreased ABTS^•+^ activity. Regarding the interaction irrigation × cultivar, for Cisco walnuts, ABTS^•+^ and FRAP were less affected under SDI_33_, and DPPH^•^ was less affected under the intermediate deficit irrigation treatments with respect to the control walnuts. A similar pattern was determined with the interaction irrigation × cv. Chandler. Consequently, these results highlight how the SDI strategies did not significantly affect the walnuts’ AA, maintaining their levels with respect to well-watered trees, thus promoting the water-saving programs.

Conversely, the total phenolic content (TPC) was significantly influenced (*p* < 0.001) by both the SDI strategy and cultivar. Notably, the SDI_50_ treatment resulted in the highest TPC value (39.1 g kg^−1^), indicating a marked enhancement under this condition. Comparable to the AA, a higher content of TPC was determined for cv. Cisco walnuts. This pattern is in line with Okatan et al. [84]; however, surprisingly, lower TPC values were determined for the Chandler and Cisco cultivars (6.90 and 7.96 g kg^−1^, respectively), which, presumably, could be ascribed to specific local conditions [85,86].

In relation to the interaction irrigation × cv. Cisco, SDI_50_ maintained the TPC level with respect to C_100_; however, it was reduced under the remaining SDI strategies. By contrast, the interaction irrigation × cv. Chandler showed that all SDI strategies augmented the TPC content. The total phenolic content (TPC) observed in both walnut cultivars in the present study exceeded the values reported by Neveu et al. [87], who documented a TPC of 15.76 g GAE kg^−1^. As highlighted by Bolling et al. [83], walnuts possess the most diverse phenolic composition and the highest phenolic content among tree nuts. Their findings indicated that the TPC in walnuts is approximately twice that of hazelnuts and four to six times greater than that of almonds, a trend that aligns with the results obtained in this study. The AA and TPC are parameters of great importance for health properties [22]. In this context, Torabian et al. [88] stated that the acute bioavailability of polyphenols from walnuts and almonds, as well as a concomitant reduction in plasma lipid peroxidation, increased the antioxidant capacity. Ojeda-Amador et al. [89], when studying three walnut varieties, one of which was Chandler, determined a TPC between 10.04 and 12.47 g GAE kg^−1^, which contributed to an AA between 105 and 170 mmol kg^−1^ for DPPH•. These values are lower for the TPC (33.6 g GAE kg^−1^) and AA (197 mmol kg^−1^) than those registered in this experiment. However, our values doubled in comparison to those of Wu et al. [90], who determined a TPC of 15.5 g GAE kg^−1^, and they were similar to those reported by Tapia et al. [12], who reported a TPC ranging from 28.0 to 58.0 g GAE kg^−1^ depending on the studied cultivar, with a content of 51.0 g GAE kg^−1^ for cv. Chandler. Furthermore, Christopoulos and Tsantili [91] revealed a content of 22.0 g GAE kg^−1^ dry weight for Chandler walnuts.

In summary, comparing the antioxidant activity (AA) and total phenolic content (TPC) values across peer-reviewed studies is challenging due to variations in extraction solvents and reference standards. Nevertheless, the findings of the present study suggest that deficit irrigation had no significant effect on the AA and TPC in the monitored walnut cultivars, except for the TPC, which showed a notable increase under the SDI_50_ treatment.

#### 2.3.3. Sugars and Mineral Contents

The impact of the SDI strategies and walnut cultivar on the sugar content for the monitored seasons is shown in Table 4. Regarding the sugars, sucrose was reduced under SDI_75_ and SDI_33_, glucose increased under SDI_75_, and the sum of the total sugars was significantly decreased under SDI_33_ with respect to C_100_. In this context, a similar pattern was highlighted by Calvo et al. [92], who subjected the Chandler walnut to three irrigation regimes based on 100, 75, and 50% of ET_C_, obtaining sucrose contents of 18.5 ± 5.02, 23.91 ± 4.49, and 22.31 ± 5.20 g kg^−1^, respectively. Additionally, Chen et al. [93] reported that the total sugar content in walnut kernels was significantly higher under deficit irrigation compared to conventional and over-irrigated treatments.

The predominance of sucrose as the main sugar in tree nuts, as observed in the present experiment, is consistent with the findings reported in previous studies [94]. The contents determined for both studied cultivars are in the range of those reported by Kazankaya et al. [95] for glucose and sucrose contents in walnut genotypes, ranging from 1.3 to 62.6 and from 17.6 to 41.7 g kg^−1^, respectively. The total sum of sugars for the monitored walnut kernels averaged 40.3 g kg^−1^, which was higher than those reported by Mitrovic et al. [96], who registered an average of 29.3 g kg^−1^.

In relation to the interactions, for irrigation × cv. Cisco, sucrose decreased under the SDI_75_ and SDI_33_ strategies, but glucose and the total sum of sugars decreased under SDI_50_ and SDI_33_, respectively. The interaction irrigation × cv. Chandler showed a reduction under all SDI levels for sucrose and under the SDI_50_ and SDI_33_ strategies for glucose and the total sum of sugars.

As observed, glucose was highly cultivar-dependent. In general, regarding the interaction irrigation × cultivar, both walnuts were affected by deficit irrigation, although some of them maintained levels similar to the control; this suggests the need to optimize the applied water stress and thus promote water savings without significant alterations.

Significant effects (*p* < 0.001) of both the SDI treatments and cultivar on the concentrations of P, K, Mn, Fe, Cu, and Zn were observed (Table 5). Notably, all water-stressed walnuts—particularly those subjected to the SDI_33_ treatment—exhibited significantly higher levels of K, Fe, and Zn. These findings suggest that SDI strategies may offer a nutritional advantage by enhancing the accumulation of certain essential minerals. The potassium (K) content observed under SDI_75_ and SDI_50_ treatments (1446–1402 mg per 100 g), and particularly under SDI_33_ (1546 mg per 100 g), may be considered a significant dietary source of this mineral. These values represent over 70% of the recommended daily allowance (2000 mg per 100 g), as outlined in Annex 1 of Council Directive 90/496/EEC [97]. In addition, Cindrić et al. [98] reported Ca, K, and Mg contents of 1062, 2771, and 1426 mg kg^−1^, respectively, in walnuts, which are lower than those found in this experiment for K and Mg and higher for the Ca content.

Regarding the micro-nutrients, there was a marked reduction for Mn and Cu due to the SDI strategies, in contrast to the Fe and Zn contents. Similarly, Carbonell-Barrachina et al. [99] reported increased Zn concentrations in tree nuts produced under water stress conditions. In general, for this study, these minerals decreased in content in the following order: Mn > Cu > Zn > Fe, which is similar to those highlighted by Cindrić et al. [98], who reported an order of Mn > Fe > Zn > Cu in walnut kernels. In addition, Antora et al. [14] reported higher contents of Fe (40 mg kg^−1^) and Zn (30 mg kg^−1^) for walnut kernels than those found in the present experiment.

On the other hand, the cultivar factor showed that cv. Cisco had significantly higher P, Fe, and Zn contents and that cv. Chandler had higher K, Mn, and Cu contents. The interaction of deficit irrigation × cv. Cisco registered a general improvement for K, Fe, and Zn and reductions for P, Mn, and Cu. The interaction irrigation × cv. Chandler augmented the K content under SDI_33_ and Fe and Zn under SDI_50_, with significant declines in the P, Mn, and Cu contents under the SDI_75_ strategy.

#### 2.3.4. Walnut Fatty Acid Profile

Table 6 illustrates the significant influence of SDI strategies and cultivar on the fatty acid profile over the monitored seasons. Specifically, the walnut lipid fraction is composed primarily of monounsaturated fatty acids (MUFAs), with oleic acid as the predominant component, and polyunsaturated fatty acids (PUFAs), mainly linoleic acid. Saturated fatty acids (SFAs), primarily palmitic and stearic acids, are also present in substantial concentrations. The predominance was as follows: linoleic > oleic > palmitic > stearic (decreasing content); other fatty acids with relevant concentrations were α-linolenic and *cis*-vaccenic, and these concentrations in walnuts were higher than those reported for almonds [100].

Regarding the impact of deficit irrigation on fatty acids, significant increases (*p* < 0.001) in the concentrations of palmitic, stearic, and linoleic acids were observed under the SDI_75_ treatment. By contrast, oleic acid was reduced under all SDI treatments in relation to the control walnuts. For most of the studied fatty acids, there were either remarkable improvements or similar concentrations under the SDI_75_ strategy with respect to the control, with reductions under the remaining SDI levels due to an increase in the water stress level. The fatty acids were also significantly affected by the cultivar, with higher concentrations for cv. Cisco than cv. Chandler, with the exception of capric, *cis*-heptadecenoic, arachidic, and α-linoleic acids. Okatan et al. [84] reported a higher content for cv. Cisco than cv. Chandler for α-linolenic, oleic, and stearic acids, which coincided with the findings of the present study, with the exception of palmitic and linoleic acids. Thus, in this study, for specific water stress levels, the content of fatty acids increased, except for oleic acid. This pattern was similar to that found by Calvo et al. [92], reporting the following for 100, 75, and 50% ET_C_ deficit irrigation treatments: palmitic (1.66, 1.75, and 1.92 g kg^−1^, respectively), linolenic (1.48, 1.76, and 1.95 g kg^−1^, respectively), oleic (1.09, 1.54, and 1.48 g kg^−1^, respectively), and stearic acids (0.93, 1.17, and 1.04 g kg^−1^, respectively). For cv. Chandler, the fatty acids in the present experiment are within the intervals reported by Rébufa et al. [101]: palmitic (2.4–69.5 g kg^−1^), stearic (7.0–34.1 g kg^−1^), oleic (61.0–178.4 g kg^−1^), linoleic (541–662 g kg^−1^), and α-linolenic acids (85.1–250.0 g kg^−1^). Martínez and Maestri [102] investigated eight walnut cultivars, including cv. Chandler, reporting considerable variation in unsaturated fatty acid concentrations: oleic acid ranged from 161 to 254 g kg^−1^, linoleic acid ranged from 525 to 589 g kg^−1^, and linolenic acid ranged from 114 to 165 g kg^−1^. This notable variability has been linked to geographic origin, with multiple studies [103,104,105] highlighting strong correlations between fatty acid profiles and the cultivation environment. In particular, the oleic acid content appears to be closely associated with climatic factors, especially air temperature, in the growing region [106]. The substantial linoleic acid content observed in the present study (253–311 g kg^−1^) aligns with findings by Ojeda-Amador et al. [89], who emphasized that the significant nutritional value of walnuts is attributed to their high linoleic acid levels, reported to range between 600 and 620 g kg^−1^. Similarly, Cittadini et al. [107] found that linoleic acid predominated (522–609 g kg^−1^) in Chandler walnut oils, whereas oleic acid was predominant in hazelnut oils (784–844 g kg^−1^). Furthermore, Pereira et al. [108] registered contents that ranged from 560 to 600, 160 to 200, and 130 to 170 g kg^−1^ for linoleic, oleic, and α-linolenic acids, respectively.

Ratios, Fatty Acid Classes, and Their Benefits

The Ω6:Ω3 ratio was significantly reduced by increasing the water deficit to SDI_50_ and SDI_33_ in comparison to the SDI_75_ and C_100_ walnuts. Correspondingly, the oleic/linoleic and Ω6:Ω3 ratios were higher for cv. Cisco walnuts. However, the decrease in the oleic/linoleic acid ratio observed under the SDI_75_ treatment may render walnuts more susceptible to reduced oil stability. It is well-established that an imbalanced dietary Ω6:Ω3 ratio, ideally maintained between 1:1 and 4:1, can negatively impact health, with some diets exhibiting ratios as high as 15:1. Such imbalances have been linked to increased risks of cardiovascular disease, autoimmune disorders, rheumatic diseases, diabetes, cancer, obesity, asthma, and depression [109,110]. Notably, walnuts are recognized for their favorable Ω6:Ω3 ratio, approximately 4:1, as reported by Zec and Glibetic [111] and Özcan et al. [112].

The predominant lipid composition based on decreasing content was SFAs < MUFAs < PUFAs, which is one of the most important characteristics of walnuts and offers health benefits (Table 7). Following the same pattern, Nogales et al. [17] reported higher values for SFAs, MUFAs, PUFAs, and Σ FAMEs of 88.7, 144.4, 766.9, and 655.8 g kg^−1^, respectively, for the Chandler cultivar. These differences can be attributed to the influence of the harvesting year, cultivation techniques, altitude, and different environmental conditions, which may influence the nut chemical composition [85,86,113]. As a whole, SDI_75_ significantly increased the SFA and PUFA contents or maintained the MUFA content with respect to the walnuts from control trees (C_100_), registering a decline when the level of water stress increased (SDI_50_ and SDI_33_). In the same way, the SDI_75_ treatment improved FAME concentrations with respect to C_100,_ with higher concentrations for cv. Cisco than cv. Chandler. Previous research has identified polyunsaturated fatty acids (PUFAs) as the predominant fatty acids in walnuts, with reported concentrations ranging from 652 to 757 g kg^−1^ [114,115,116]. In the present study, the highest PUFA content in extracted oils was 386 g kg^−1^, observed in samples under the SDI_75_ treatment, further confirming that walnuts contain substantial amounts of PUFAs. The increase in PUFA levels under deficit irrigation has also been noted in other tree nut studies [99,100]. These results are consistent with the findings of Gutiérrez et al. [117], who demonstrated that deficit irrigation can enhance the monounsaturated fatty acid (MUFA) content in nut crops such as almonds. The PUFA:SFA ratio is a widely used index to assess the impact of diet on cardiovascular health and to evaluate the nutritional quality of foods. A higher ratio is generally considered beneficial, as supported by Chen and Liu [118]. According to EFSA [119], linoleic acid plays a crucial role in cardiac cell function and is an essential Ω6 polyunsaturated fatty acid, which is important for human health. The recommended daily intake for linoleic acid is 10 g, and consumption of approximately 25 g of walnuts produced under the SDI_75_ treatment, particularly the Cisco cultivar, can provide nearly 78% of this requirement.

Conversely, the atherogenic index, which indicates the potential of a diet to promote coronary diseases, showed a significant difference only under the SDI_75_ treatment, while the cultivar factor had no significant effect on this index. Similarly, the thrombogenic index, which reflects the tendency for clot formation in blood vessels, was elevated in walnuts subjected to SDI_75_.

It is important to note that, depending on the severity of water deficit, there may be an economic threshold beyond which deficit irrigation is insufficient to sustain walnut tree growth and yield potential. Prolonged water stress can thus lead to considerable economic losses. However, one potential strategy to offset this loss is the production of walnuts with enhanced value through improved quality, particularly via increased levels of bioactive compounds.

### 2.4. Descriptive Sensory Analysis

The descriptive sensory analysis for the external appearance and internal properties of the studied walnut cultivars in relation to the effect of SDI is shown in Table 7. Overall, significant differences were detected in 7 of the 15 attributes used to evaluate walnut quality. Specifically, the panelists noted that all SDI treatments led to reductions in the outer color, size, vein prominence, overall nut appearance, and floral/fruity aroma, with these effects being more pronounced in walnuts subjected to severe water stress (SDI_33_) compared to the control group. However, the values could be maintained similar to control walnuts if SDI_75_ is applied, with the exception of veins and floral/fruity attributes. According to the sensorial analysis by the panelists, the sweetness was not affected under SDI_33_, but a slight increase (4.6 vs. 5.1) was recorded for SDI_50_ and SDI_75_. In this context, sweetness is a key attribute influencing the sensory quality of walnuts, and an increase in its intensity is expected to enhance consumer satisfaction. The cv. Chandler was significantly (*p* < 0.001) sweeter than cv. Cisco. In addition, the astringency increased with all SDI strategies, and the woody parameter especially increased with SDI_75_. In this context, the results by Ingels et al. [120] showed no differences in astringency among cultivars; however, cv. Chandler was judged to be sweeter than the other studied cultivars (Howard and Chico). All sensory properties related to appearance were evaluated visually, encompassing both color and geometric attributes, which are essential parameters, as highlighted by Shepherd et al. [121].

Overall, the results for color and size were consistent with the findings presented in Table 1, although variations in color assessments may arise due to the inherent difficulty of perceiving subtle details with the human eye. The results of the sensory size were similar to the instrumental size determined and discussed before. The outer color, veins, overall nuts, walnut ID, and woody attributes were significantly higher for cv. Cisco, and the size, sweetness, bitterness, and aftertaste were higher for cv. Chandler. Between the two evaluated walnuts, Cisco was less bitter than Chandler; this characteristic makes cv. Cisco kernels more marketable, and this could be appreciated by consumers. In addition, Sinesio and Moneta [122] highlighted that walnut bitterness and astringency descriptors appeared to be positively correlated, which can be significant in walnut quality perception by consumers. In contrast, Peleg et al. [123] revealed that the main factor that affected the sensory properties of bitterness and astringency was the molecular size of volatile flavonoids; as the molecular size increased, the bitterness decreased and the astringency increased.

In relation to the interaction irrigation × cv. Cisco, higher values under the SDI_75_ and SDI_50_ strategies were found for the outer color, size, sweetness, and overall nuts, while under SDI_33,_ there were higher values for bitterness and astringency. Walnut ID and aftertaste significantly declined in walnuts from SDI_33_ plots. Regarding the interaction irrigation × cv. Chandler, water stress significantly decreased the values of the outer color and size, particularly under SDI_33_, and the overall nuts, walnut ID, and floral/fruity attributes decreased under SDI_75_. By contrast, the bitterness and astringency were augmented under the SDI_75_ strategy.

These results offer valuable insights into the sensory properties of the cultivars studied under deficit irrigation conditions. Based on the findings, it is recommended that consumer preferences be taken into account during cultivar selection, considering not only the walnut variety but also the level of water stress applied throughout the production process before making final decisions.

## 3. Materials and Methods

### 3.1. Study Area

The study area is located within the agroforestry watershed known as ‘El Salado,’ situated in the Sierra Nevada Mountains near Lanjarón, Granada, in southeastern Spain. This watershed typifies a mid-altitude Mediterranean mountain environment, characterized by a mix of rainfed and irrigated crops, alongside reforested pine stands. The mean annual precipitation in the study area is approximately 531 mm, exhibiting considerable inter-annual variability. Most rainfall is concentrated in the winter and autumn seasons, while intense short-duration storms are common in spring but infrequent during summer. The average annual, maximum, and minimum temperatures are 15.0 °C, 20.8 °C, and 9.2 °C, respectively.

At the study site, the dominant soil parent material consists of colluvium and residuum derived from mica-schist, with a weathered regolith layer only a few centimeters deep. The soils are well-drained and classified as *Eutric Cambisols* [124]. The soil texture comprises 654 g kg^−1^ sand, 250 g kg^−1^ silt, and 96 g kg^−1^ clay. The soil pH is 7.6 (1:2.5 soil-to-water ratio), and the bulk density is 1.16 g cm^−3^. The soil organic carbon and total nitrogen contents are 10.0 and 0.60 g kg^−1^, respectively, while extractable P (Olsen method) and available N concentrations are 6.3 mg kg^−1^ and 78.4 mg kg^−1^, respectively. The experimental walnut orchard, located at an elevation of 670 m a.s.l., is established on terraced land and consists of 39-year-old trees from two cultivars, namely, Chandler and Cisco. The trees are healthy and of uniform size and were planted at 6-m intervals, resulting in approximately 272 trees per hectare. Uniform fertilization was applied throughout the orchard, with each tree receiving 280 g N, 195 g P₂O₅, and 242 g K₂O. Standard local management practices, including pruning, weed control, and pest and disease management, were consistently implemented across the entire experimental area, alongside the irrigation treatments.

### 3.2. Irrigation Strategies and Experimental Design

Three sustained deficit irrigation (SDI) regimes were applied, supplying 33% (SDI_33_), 50% (SDI_50_), and 75% (SDI_75_) of the crop evapotranspiration (ET_C_) requirements. A control treatment (C_100_) was also included, in which crops received full irrigation at 100% ET_C_ to meet the entire evapotranspiration demand throughout the irrigation period.

To estimate the irrigation requirements, reference evapotranspiration (ET₀) was calculated using the Penman–Monteith method, based on data obtained from a weather station located within the experimental walnut orchard. Crop coefficient (Kc) values were determined according to the guidelines outlined by Allen et al. [125]. The irrigation system implemented for each treatment included two lateral drip lines per tree row, positioned 50 cm from either side of the trunk. Each line was equipped with a combination of pressure-compensating drip emitters delivering flow rates of 4 and 8 L h^−1^, spaced at 50 cm intervals. In general, the experimental plots were irrigated on alternate days from May to September. During the peak evaporative demand period (June to August), irrigation was applied daily for 4–5 h. Following this period, in September, the irrigation frequency was reduced to one or two times per week, with each session lasting 2–3 h. The irrigation reductions according to each SDI strategy were applied uniformly throughout the growing season across all tree walnut development phases. Water stress was induced by reducing the water irrigation doses, with the average irrigation per season during the study period for the C_100_, SDI_75_, SDI_50_, and SDI_33_ plots amounting to 6723 ± 346, 5042 ± 435, 3361 ± 387, and 2218 ± 214 m^3^ ha^−1^, respectively.

The experimental design was a completely randomized block design with three replications per irrigation treatment (four trees per experimental unit plot with an area of about 70 m^2^); the two central trees from each row were included for fruit yield measurements, and the others served as border trees. Harvesting operations were performed using a mechanical vibrator; later, walnuts were air-dried and weighed once they reached a humidity content below about 6%. At the end of each season, walnut production was measured in terms of the in-shell yield for SDI treatments as well as for control trees, and the water productivity (WP) for each irrigation strategy was estimated. From these harvested nuts, kernel samples of each replication and irrigation treatment were selected for quality and descriptive sensorial analyses.

### 3.3. Physical and Chemical Walnut Nut Analysis

#### 3.3.1. Morphological, Moisture Content, Instrumental Color, Cutting Force, and Water Activity Measurements

Walnut samples were randomly selected and analyzed through the determination of the kernel weight and dimensions (length and width). The kernel weight was measured using an analytical balance (Mettler Toledo model AG204, Barcelona, Spain), while size measurements were obtained with a digital caliper (Mitutoyo 500-197-20, Kawasaki, Japan). The data were determined as the mean of 20 repetitions. The moisture content was determined using the standard method [126]; for this, 2 g of a ground sample was placed on a metallic tray and dried in an oven at 60 °C until a constant mass was achieved. The color of the walnut kernels was determined using a Minolta Colorimeter CR-300 (Minolta, Osaka, Japan), using CIEL*a*b* coordinates, defined as three numerical values in a three-dimensional space. For this analysis, twenty measurements were made on each sample; the values for L*, a*, and b* were averaged, and from these data, the hue values were estimated. The cutting force of walnuts was analyzed using a texture analyzer (Stable Micro Systems, model TA-XT2i, Godalming, UK) loaded with a 30 kg cell and a probe (Volodkevich Bite Jaw HDP/VB, Stable Micro Systems, Godalming, UK). The trigger was set to 15 g, with a test displacement rate of 1 mm s^−1^ over 3 mm, to determine two parameters, namely, hardness (N) and number of fractures (peak count), as the mean value of twenty measurements. Finally, the water activity (a_W_) was determined using a meter device (Novasina AW-SPRINT TH500; Pfaffikon, Zurich, Switzerland).

#### 3.3.2. Antioxidant Activity and Total Polyphenol Determination

Samples of ground walnuts were used for the extraction of the antioxidant activity (AA) and of polyphenols using a methanolic extractant (MeOH:H_2_O; 80:20; *v*:*v* + 1% HCl). The AA, as mmol of Trolox kg^−1^ in walnuts, was determined using ABTS^•+^ (2,2′-azino-bis(3-ethylbenzthiazoline-6-sulphonic acid) according to the methodology described by Re et al. [127], and DPPH^•^ (2,2-diphenyl-1-picryl-hydrazyl-hydrate) radicals were measured according to the procedure described by Brand et al. [128]. Additionally, AA was also measured by the ability to reduce iron ions (FRAP) [129]. All measurements were made using an ultraviolet–visible (UV–Vis) spectrophotometer (Helios Gamma model, UVG 1002E; Helios, Cambridge, UK).

The total phenolic content (TPC) was determined using the Folin–Ciocalteu colorimetric method, according to Gao et al. [130]. The walnut extract (0.1 mL) was mixed with the Folin–Ciocalteu reagent (0.2 mL) and 2 mL of distilled water. After 3 min, 1 mL of a 20% aqueous solution of sodium carbonate (Na_2_CO_3_) was added. The absorbance at 765 nm was measured after 1 h. The results are presented as g of gallic acid equivalent (GAE) kg^−1^.

#### 3.3.3. Sugar and Fatty Acid Methyl Ester Determination

The sugar profile was analyzed using high-performance liquid chromatography (HPLC). The extraction consisted of the homogenization of 1 g of ground walnuts with 5 mL of phosphate buffer (pH = 7.8), and a sample was filtered (0.45 µm Millipore membrane filter) and injected into a Hewlett Packard (Wilmington, DE) series 1100 HPLC. For the elution buffer, 0.1% orthophosphoric acid was used. For the separation of compounds, a Supelcogel TM C-610H column (30 cm × 7.8 mm) with a pre-column (Supelguard 5 cm × 4.6 mm; Supelco, Bellefonte, PA, USA) was used. The sugar contents were measured using a refractive index detector.

The fatty acid methyl esters (FAMEs) were determined using methylation, as described by Lipan et al. [131], with some modifications. Methyl esters of fatty acids were separated in a Shimadzu GC17 (Shimadzu Corporation, Kyoto, Japan), a gas chromatograph coupled with a flame ionization detector and a DB-23 capillary column (Agilent Technologies, Santa Clara, California, United States). The carrier gas was He with an initial flow rate of 1.2 mL min^−1^, while the detector gases were H**_2_** (30 mL min^−1^) and air (350 mL min^−1^), with He (35 mL^−1^) as the make-up gas. The injector and detector temperatures were 250 and 260 °C, respectively, with an injection volume of 0.6 µL and a split ratio of 1:10. The preliminary temperature was 175 °C for 10 min, with a temperature gradient of 3 °C min^−1^ to 215 °C, and 215 °C was retained for 15 min. The identification of FAME peaks was conducted by comparing the results with the retention times of the standards (FAME Supelco MIX-37, Merck KGaA, Darmstadt, Germany). The contents are presented in g kg^−1^, with methyl nonadecanoate as the internal standard.

#### 3.3.4. Mineral Content Determination

The mineral contents were determined using a microwave digestion unit, namely, Ethos Easy, Milestone (Milestone, Sorisole, Italy), equipped with a rotor for ten TFM (chemically modified PTFE) vessels for sample mineralization and an inductively coupled plasma mass spectrometry (ICP-MS) instrument, namely, Agilent 7500× Octopole Reaction System (ORS) (Agilent Technologies, Tokyo, Japan), for mineral determination, as described by Cano-Lamadrid et al. [132]. The measurements were conducted in lyophilized samples, and the results (mean of 4 replications) are presented as mg kg^−1^ of dried matter.

#### 3.3.5. Descriptive Analysis

**Figure 3:**
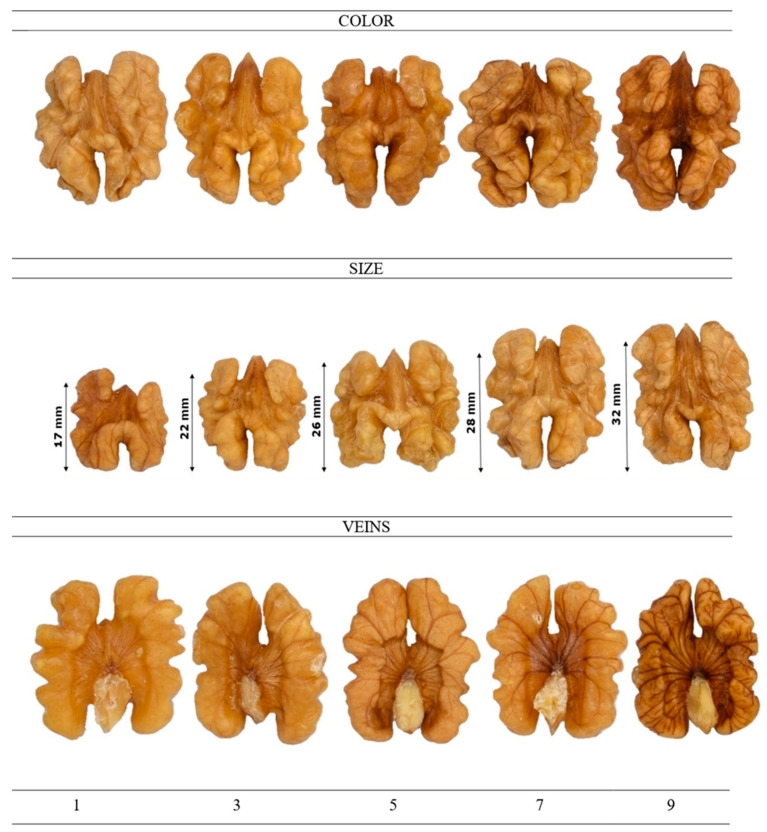
Reference scales for the descriptive sensory analysis of the walnut’s appearance. Numerical scale, where 1 means “very weak” and 10 means “very strong”.

The descriptive analysis was carried out by a trained panel, consisting of 10 highly qualified panelists from the Food Quality and Safety Group (Miguel Hernández University of Elche, Orihuela, Alicante, Spain). The descriptive sensory analysis was performed according to the method described by Lipan et al. [100] to estimate the differences among SDI treatments. The walnut samples were served in an odor-free 30 mL covered plastic cup and randomly coded with three digits. Water and unsalted crackers were served in order to cleanse the palate between samples. The samples of walnuts were presented based on a randomized block design to avoid biases. A numerical scale from 0 to 10, with 0.5 increments, was used by the panelists to quantify the intensity of the walnut attributes, where 0 represents no intensity and 10 means extremely strong. The reference products used by the panelists for flavor and texture characterization are shown in Table 8, and Figure 3 displays the reference scales for the descriptive sensory analysis of walnuts.

### 3.4. Statistical Analysis

Walnut yield was analyzed using Tukey’s least significant difference test (*p* < 0.05). The fruit quality parameters were analyzed using two-way analysis of variance (ANOVA) to characterize the data; then, the data were subjected to Tukey’s multiple range test. XLSTAT Premium 2016 (Addinsoft, New York, NY, USA) was used to calculate statistically significant differences, with significance levels of *p* < 0.05, *p* < 0.01, and *p* < 0.001.

## 4. Conclusions

This study highlights the environmental, agronomic, and potential health benefits for walnut consumers by implementing deficit irrigation within sustainable intensification frameworks for walnut production. Concretely, we showed the advantageous effect of SDI on walnut tree performance, as it enhances the water productivity and reveals walnut’s tolerance to moderate and medium water deficits, particularly cv. Cisco. The experiment demonstrated that reducing the irrigation water by 25% and 50% resulted in yield reductions of 21% and 39%, respectively, accompanied by an increase in WP. A 67% reduction in irrigation produced variable yield effects depending on the cultivar; however, WP generally reached its highest levels compared to fully irrigated control trees. Nonetheless, severe deficit irrigation (SDI_33_) caused a significant yield reduction of approximately 50%. Considering water savings alongside potential economic returns, factoring in the yield and fruit size, the SDI_75_ treatment emerges as the most favorable irrigation strategy, balancing acceptable yield losses (21% relative to non-water-stressed trees) with efficient water use.

The effects of deficit irrigation on the morphological and physicochemical characteristics were particularly pronounced under the severe SDI_33_ treatment. Conversely, the SDI_75_ strategy appears to be a viable approach for optimizing the walnut lipid profile, as it enhanced the concentrations of essential fatty acids such as Ω3 and Ω6 as well as other unsaturated fatty acids, all of which hold significant dietary value. However, the SDI_50_ and SDI_33_ treatments were associated with substantial reductions in productivity compared to SDI_75_. Therefore, SDI_75_ represents the most suitable irrigation regime, achieving a sustainable balance among fruit yield, quality, and water conservation. Accordingly, walnuts from moderately water-stressed trees may offer superior health benefits, particularly due to their elevated levels of bioactive phytochemicals.

## Figures and Tables

**Table 1:** Morphology, instrumental color, and instrumental texture of raw walnuts as affected by irrigation treatment and walnut cultivars during the monitoring period.

	Weight (g)			Size (mm)			Instrumental Color					Cutting Force	
	Whole	Kernel	Shell	Length	Width	Thickness	L*	a*	b*	C	Hue	Hardness (N)	NF
	ANOVA Test ^†^												
Irrigation	***	***	***	***	***	***	**	***	NS	NS	***	***	***
Cultivar	***	NS	***	NS	***	NS	***	***	***	***	***	***	***
Irrigation × Cultivar	***	***	***	***	***	***	**	***	***	***	***	***	***
	Tukey’s Multiple Range Test ^‡^												
Irrigation													
C_100_	10.20 a	4.55 a	5.64 a	36.7 a	32.6 a	30.7 a	53.8 b	7.63 a	28.8	29.9	75.2 b	28.4 b	32.9 bc
SDI_75_	9.08 bc	4.06 b	5.02 ab	35.7 a	31.0 b	29.4 b	55.5 a	7.23 a	28.9	29.9	76.0 a	33.7 a	29.2 c
SDI_50_	9.35 ab	4.11 ab	5.24 ab	35.5 a	31.3 b	29.4 b	55.2 a	7.66 a	29.7	30.7	75.5 b	28.7 b	41.9 a
SDI_33_	8.22 c	3.53 c	4.68 b	32.9 b	29.7 c	28.2 c	56.5 a	6.84 b	29.2	30.1	76.9 a	29.3 b	38.4 ab
Cultivar													
Cisco	9.59 a	4.08	5.51 a	35.2	31.5 a	29.3	56.3 a	8.16 a	30.6 a	31.7 a	75.0 b	29.0 b	36.9 a
Chandler	8.83 b	4.05	4.78 b	35.2	30.9 b	29.6	54.4 b	6.58 b	27.9 b	28.7 b	76.7 a	31.1 a	34.3 b
Irrigation × Cultivar													
C_100_ × Cisco	10.5 a	4.61 a	5.86 a	36.5 a	33.1 a	30.6 a	54.8 b	8.90 a	31.7 a	33.0 a	74.1 b	26.4 c	35.0 b
SDI_75_ × Cisco	9.58 b	4.06 a	5.52 a	35.8 ab	31.2 b	29.3 ab	57.9 a	7.54 b	30.1 a	31.1 b	75.9 a	32.2 b	29.2 c
SDI_50_ × Cisco	10.5 a	4.51 a	6.04 a	36.7 a	32.9 a	30.4 a	54.3 b	8.97 a	30.4 a	31.7 b	73.5 b	26.6 c	42.0 a
SDI_33_ × Cisco	7.75 d	3.13 c	4.62 b	32.0 c	28.7 c	26.8 c	57.8 a	7.44 b	30.4 a	31.4 b	76.3 a	30.8 b	41.4 a
C_100_ × Chandler	9.91 b	4.49 a	5.42 a	36.9 a	32.1 a	30.7 a	53.0 c	6.66 c	26.7 d	27.5 d	76.0 a	30.5 b	30.8 c
SDI_75_ × Chandler	8.57 c	4.06 a	4.51 b	35.6 ab	30.9 b	29.5 ab	53.4 c	6.94 c	27.8 c	28.7 d	76.0 a	35.2 a	29.1 c
SDI_50_ × Chandler	8.15 c	3.72 b	4.43 b	34.3 b	29.7 b	28.5 b	56.0 b	6.47 c	29.1 b	29.8 c	77.4 a	30.8 b	41.8 a
SDI_33_ × Chandler	8.69 c	3.93 ab	4.75 b	33.9 bc	30.8 b	29.5 ab	55.3 b	6.24 c	28.0 c	28.8 d	77.5 a	27.9 c	35.5 b

SDI, sustained deficit irrigation; SDI_33_, at 33% ET_C_; SDI_50_, at 50% ET_C_; SDI_75_, at 75% ET_C_; and C100, control at 100% ET_C_. ^†^ NS = not significant; ** and *** = significant at *p* < 0.01 and 0.001, respectively. ^‡^ Values associated with different letters within the same column and factor were significantly different (*p* < 0.05) according to Tukey’s least significant difference test. L*, a*, and b* = color coordinates; C = chroma; and NF = number of fractures.

**Table 2:** Effect of irrigation dose and cultivar on the walnut kernel ratio, moisture content, and water activity during the study period.

	Kernel Ratio	Dry Weight	Water Activity
	(%)		(aw)
	ANOVA Test ^†^		
Irrigation	***	***	***
Cultivar	NS	NS	NS
Irrigation × Cultivar	***	***	***
	Tukey’s Multiple Range Test ^‡^		
Irrigation			
C_100_	39.0 a	6.59 a	0.72 a
SDI_75_	40.7 a	3.26 c	0.61 b
SDI_50_	29.5 b	5.49 ab	0.67 a
SDI_33_	38.0 a	4.76 b	0.60 b
Cultivar			
Cisco	36.1	5.23	0.66
Chandler	37.5	4.82	0.64
Irrigation × Cultivar			
C_100_ × Cisco	38.1 ab	7.23 a	0.78 a
SDI_75_ × Cisco	36.7 ab	2.97 d	0.61 bc
SDI_50_ × Cisco	34.0 bc	5.17 abcd	0.68 b
SDI_33_ × Cisco	35.5 abc	5.56 abc	0.58 c
C_100_ × Chandler	39.9 ab	5.95 ab	0.66 bc
SDI_75_ × Chandler	44.6 a	3.55 cd	0.60 bc
SDI_50_ × Chandler	25.0 c	5.81 abc	0.67 bc
SDI_33_ × Chandler	40.5 ab	3.96 bcd	0.61 bc

SDI, sustained deficit irrigation; SDI_33_, at 33% ET_C_; SDI_50_, at 50% ET_C_; SDI_75_, at 75% ET_C_; and C_100_, control at 100% ET_C_. ^†^ NS = not significant; *** = significant at *p* < 0.001. ^‡^ Values followed by different letters within the same column and factor were significantly different (*p* < 0.05) according to Tukey’s least significant difference test.

**Table 3:** Antioxidant activity and total phenolic content of walnuts as affected by deficit irrigation and cultivar factors.

	ABTS^•+^	DPPH^•^	FRAP	TPC
	(mmol Trolox kg^−1^)			(g GAE kg^−1^)
	ANOVA Test ^†^			
Irrigation	***	***	***	***
Cultivar	***	***	***	***
Irrigation × Cultivar	***	***	***	***
Irrigation	Tukey’s Multiple Range Test ^‡^			
C_100_	140 a	227 a	149 a	35.6 b
SDI_75_	122 b	221 a	141 b	36.4 b
SDI_50_	115 b	226 a	148 ab	39.1 a
SDI_33_	138 a	195 b	152 a	36.4 b
Cultivar				
Cisco	148 a	237 a	159 a	40.2 a
Chandler	110 b	197 b	135 b	33.6 b
Irrigation × Cultivar				
C_100_ × Cisco	164 a	254 a	168 ab	42.6 a
SDI_75_ × Cisco	136 b	248 a	136 cd	37.2 b
SDI_50_ × Cisco	131 b	250 a	159 b	42.2 a
SDI_33_ × Cisco	162 a	196 b	174 a	38.7 b
C_100_ × Chandler	117 c	200 b	130 d	28.7 d
SDI_75_ × Chandler	109 d	194 b	145 c	35.6 c
SDI_50_ × Chandler	98.3 d	201 b	136 cd	35.9 c
SDI_33_ × Chandler	115 c	193 b	129 d	34.0 c

SDI, sustained deficit irrigation; SDI_33_, at 33% ET_C_; SDI_50_, at 50% ET_C_; SDI_75_, at 75% ET_C_; and C_100_, control at 100% ET_C_. ABTS^•+^, 2,2′-azino-bis(3-ethylbenzthiazoline-6-sulphonic acid); DPPH^•^, 2,2-diphenyl-1-picryl-hydrazyl-hydrate; FRAP, ferric reducing antioxidant power; TPC, total phenolic content; ^†^ NS = not significant; and *** = significant at *p* < 0.001. ^‡^ Values followed by different letters within the same column and factor were significantly different (*p* < 0.05) according to Tukey’s least significant difference test.

**Table 4:** Sugar content in raw walnuts as affected by irrigation dose and cultivar.

	Sucrose	Glucose	Σ Sugars
		(g kg^−1^dw)	
		ANOVA Test ^†^	
Irrigation	***	**	**
Cultivar	NS	**	NS
Irrigation × Cultivar	***	**	**
	Tukey’s Multiple Range Test ^‡^		
Irrigation			
C_100_	27.3 a	14.8 b	42.2 a
SDI_75_	24.4 b	16.2 a	40.6 a
SDI_50_	26.6 a	13.8 b	40.4 a
SDI_33_	23.4 b	14.7 b	38.1 b
Cultivar			
Cisco	25.3	15.3 a	40.5
Chandler	25.6	14.5 b	40.1
Irrigation × Cultivar			
C_100_ × Cisco	27.9 a	15.6 ab	43.5 a
SDI_75_ × Cisco	23.6 cd	16.0 ab	39.6 ab
SDI_50_ × Cisco	27.7 a	13.8 b	41.5 a
SDI_33_ × Cisco	21.9 d	15.6 ab	37.5 c
C_100_ × Chandler	26.8 b	14.0 ab	40.8 a
SDI_75_ × Chandler	25.1 c	16.4 a	41.5 a
SDI_50_ × Chandler	25.5 c	13.8 b	39.3 b
SDI_33_ × Chandler	24.9 c	13.8 b	38.7 b

SDI, sustained deficit irrigation; SDI_33_, at 33% ET_C_; SDI_50_, at 50% ET_C_; SDI_75_, at 75% ET_C_; and C_100_, control at 100% ET_C_. ^†^ NS = not significant; ** and *** = significant at *p* < 0.01 and 0.001, respectively. ^‡^ Values followed by different letters within the same column and factor were significantly different (*p* < 0.05) according to Tukey’s least significant difference test.

**Table 5:** Mineral content in raw walnuts as affected by the irrigation dose and cultivar for the study period.

	B	Mg	P	K	Ca	Mn	Fe	Cu	Zn
	(mg kg^−1^_dw_)								
	ANOVA Test ^†^								
Irrigation	NS	NS	*	*	NS	***	***	***	***
Cultivar	NS	NS	*	*	NS	***	***	***	***
Irrigation × Cultivar	NS	NS	*	*	NS	***	***	***	***
	Tukey’s Multiple Range Test ^‡^								
Irrigation									
C_100_	14.8	10,617	35.3 a	12,940 c	7.43	475 a	5.33 b	182 a	14.8 b
SDI_75_	12.5	11,555	30.1 b	14,462 b	7.23	356 b	7.83 a	116 b	24.1 a
SDI_50_	14.2	11,450	34.3 a	14,024 b	7.42	354 b	8.66 a	107 b	25.5 a
SDI_33_	13.5	11,795	30.3 b	15,467 a	6.21	303 b	8.19 a	99 b	27.5 a
Cultivar									
Cisco	14.4	10,963	34.8 a	13,401 b	7.40	326 b	9.10 a	109 b	28.7 a
Chandler	13.1	11,746	30.2 ab	15,045 a	6.75	418 a	5.89 b	143 a	17.2 b
Irrigation × Cultivar									
C_100_ × Cisco	15.5	10,281	38.5 a	12,062 c	8.13	438 ab	6.37 bc	169 b	17.5 c
SDI_75_ × Cisco	12.7	11,867	32.6 b	13,728 b	7.83	345 b	9.92 a	110 cd	30.4 a
SDI_50_ × Cisco	15.1	10,897	37.2 a	12,969 b	7.57	323 bc	10.3 a	89.5 cd	31.4 a
SDI_33_ × Cisco	14.3	10,806	30.7 b	14,845 b	6.06	198 c	9.87 a	67.5 d	35.7 a
C_100_ × Chandler	14.0	10,952	32.0 b	13,818 b	6.73	511 a	4.28 c	195 a	12.1 c
SDI_75_ × Chandler	12.2	11,243	27.6 c	15,196 b	6.62	366 b	5.73 bc	122 c	17.9 c
SDI_50_ × Chandler	13.4	12,003	31.4 b	15,079 b	7.27	385 b	7.06 b	125 c	19.6 bc
SDI_33_ × Chandler	12.7	12,784	29.9 b	16,088 a	6.37	408 b	6.50 bc	131 c	19.3 bc

SDI, sustained deficit irrigation; SDI_33_, at 33% ET_C_; SDI_50_, at 50% ET_C_; SDI_75_, at 75% ET_C_; and C_100_, control at 100% ET_C_. ^†^ NS = not significant; * and *** = significant at *p* < 0.05, and 0.001, respectively. ^‡^ Values followed by different letters within the same column and factor were significantly different (*p* < 0.05) according to Tukey’s least significant difference test.

**Table 6:** Effect of irrigation dose and walnut cultivar on the fatty acid methyl esters (FAMEs).

Compound (FAMEs)	ANOVA Test		Irrigation				Cultivar	
	Irrigation	Cultivar	C_100_	SDI_75_	SDI_50_	SDI_33_	Cisco	Chandler
	(g kg^−1^_dw_)							
C10:0 (Capric)	***	***	0.003 c	0.005 a	0.004 b	0.003 c	0.003 b	0.005 a
C12:0 (Lauric)	***	***	0.009 b	0.012 a	0.008 c	0.008 c	0.009 b	0.010 a
C14:0 (Myristic)	***	***	0.040 c	0.053 a	0.045 b	0.051 a	0.049 a	0.046 b
C14:1 (Myristoleic)	***	***	0.071 a	0.071 a	0.067 b	0.070 ab	0.071 a	0.068 b
C15:0 (Pentadecylic)	***	***	0.036 b	0.041 a	0.038 b	0.038 b	0.039 a	0.037 b
C16:0 (Palmitic)	***	***	26.9 b	31.6 a	24.8 c	25.8 bc	27.9 a	26.6 b
C16:1c7 (*cis*-Hexadecenoic)	***	***	0.19 b	0.21 a	0.18 b	0.18 b	0.19 a	0.18 b
C16:1c9 (Palmitoleic)	***	***	0.28 b	0.31 a	0.30 a	0.29 ab	0.31 a	0.29 b
C16:1c10	***	***	0.012 d	0.018 a	0.016 b	0.013 c	0.02 a	0.01 b
C17:0 (Margaric)	***	***	0.15 b	0.19 a	0.14 b	0.15 b	0.17 a	0.14 b
C17:1 (*cis*-Heptadecenoic)	***	***	0.05 c	0.08 a	0.06 b	0.06 b	0.06 b	0.07 a
C18:0 (Stearic)	***	***	9.98 b	11.4 a	9.59 bc	9.23 c	11.3 a	8.82 b
C18:1t9 (Elaidic)	***	***	0.10 b	0.13 a	0.09 c	0.08 c	0.104 a	0.098 b
C18:1c9 (Oleic)	***	***	75.5 a	73.8 b	66.7 c	65.6 c	78.2 a	62.7 b
C18:1n7 (*cis*-Vaccenic)	***	***	3.91 b	4.21 a	3.44 d	3.63 c	3.84 a	3.75 b
C18:2 t8c13 (Linoleaidic)	***	***	0.19 b	0.25 a	0.19 b	0.20 b	0.20 b	0.21 a
C18:2n6cis 9,12 (Linoleic)	***	***	290 b	311 a	253 c	263 c	288 a	271 b
C18:3n6 (γ-Linolenic)	***	***	0.35 b	0.37 a	0.31 c	0.32 c	0.36 a	0.31 b
C20:0 (Arachidic)	***	***	0.16 c	0.27 a	0.21 b	0.22 b	0.18 b	0.26 a
C18:3n3 (α-Linolenic)	***	***	67.6 b	73.3 a	67.1 b	72.2 a	64.4 b	75.7 a
C21:0 (Heneicosanoic)	***	***	0.073 c	0.082 a	0.078 b	0.075 bc	0.079 a	0.075 b
C20:2n6 (Eicosadienoic)	**	**	0.07 b	0.10 a	0.08 b	0.09 ab	0.08 a	0.09 a
C20:3n3 (Eicosatrienoico)	***	***	0.09 b	0.21 a	0.10 b	0.10 b	0.14 a	0.12 b
C20:3n6 (Eicosatrienoico)	***	***	0.03 b	0.04 a	0.02 c	0.03 b	0.03 a	0.02 b
C23:0 (Tricosanoic acid)	***	***	0.030 a	0.030 a	0.029 ab	0.027 b	0.031 a	0.027 b
C20:5n3 (Eicosapentanoico)	***	***	0.016 b	0.019 a	0.015 bc	0.014 c	0.02 a	0.01 b
C24:0 (Lignoceric acid)	***	***	0.047 b	0.060 a	0.044 b	0.046 bc	0.05 a	0.04 b
Oleic:Linoleic	***	***	0.250 b	0.240 c	0.260 a	0.250 b	0.270 a	0.230 b
Ω 6:Ω 3	***	***	4.31 a	4.24 a	3.86 b	3.68 c	4.47 a	3.57 b
Saturated (SFA)	***	***	37.5 b	43.7 a	35.0	35.6 c	39.8 a	36.1 b
Monounsaturated (MUFA)	***	***	80.1 a	78.8 a	70.9 b	70.0 b	82.8 a	67.1 b
Polyunsaturated (PUFA)	***	***	358 b	386 a	321 d	336 c	353 a	347 a
PUFA:SFA	***	***	9.81 a	8.81 c	9.16 bc	9.46 ab	8.87 b	9.75 a
PUFA:MUFA	***	***	4.78 a	4.89 a	4.61 b	4.82 a	4.33 b	5.22 a
(MUFA + PUFA)/SFA	***	***	11.9 a	10.6 c	11.2 b	11.4 ab	10.9 b	11.6 a
Atherogenic index	***	***	0.06 c	0.07 a	0.06 b	0.06 b	0.065 a	0.064 a
Thrombogenic index	***	***	0.09 b	0.10 a	0.09 b	0.09 b	0.10 a	0.09 b
Σ FAMEs	***	***	475 b	508 a	427 d	441 c	475 a	451 b

SDI, sustained deficit irrigation; SDI_33_, at 33% ET_C_; SDI_50_, at 50% ET_C_; SDI_75_, at 75% ET_C_; and C_100_, control at 100% ET_C_. ** and *** = significant at *p* < 0.01 and 0.001, respectively; Values followed by different letters within the same row and factor were significantly different (*p* < 0.05) according to Tukey’s least significant difference test. Σ FAMEs = fatty acids methyl esters.

**Table 7:** Descriptive sensory analysis of walnuts as affected by deficit irrigation.

	Outer Color	Size	Veins	Sweetness	Bitterness	Astringency	Overall nuts	Walnut ID	Floral/Fruity	Woody	Hardness	Cohesiveness	Crispiness	Adhesiveness	Aftertaste
	ANOVA Test ^†^														
Irrigation	***	***	***	***	NS	**	***	NS	***	***	NS	NS	NS	NS	NS
Cultivar	***	***	***	***	*	NS	***	***	***	***	NS	NS	NS	NS	***
Irrigation × Cultivar	***	***	***	***	*	**	***	***	***	***	NS	NS	NS	NS	***
	Tukey’s Multiple Range Test ^‡^														
Irrigation															
C_100_	4.2 b	6.0 a	6.4 a	4.6 b	0.6	1.7 c	7.2 a	7.6	0.8 a	2.9 c	3.2	7.1	2.0	7.9	8.1
SDI_75_	4.0 b	5.8 a	2.9 c	5.1 a	0.8	2.2 b	7.2 a	7.3	0.1 b	4.2 a	4.0	6.9	2.9	7.3	7.4
SDI_50_	5.3 a	5.8 a	4.7 b	5.1 a	1.2	2.0 b	7.4 a	7.8	0.1 b	2.8 c	3.5	6.8	2.8	7.5	7.9
SDI_33_	3.1 c	3.1 b	2.3 c	4.6 b	1.1	2.6 a	6.9 b	7.5	0.4 b	3.7 b	3.6	6.8	2.6	7.4	7.8
Cultivar															
Cisco	5.2 a	4.5 b	4.6 a	4.3 b	0.7 b	2.1	8.1 a	8.3 a	nd	3.8 a	3.3	7.0	2.5	7.6	7.5 b
Chandler	3.1 b	5.9 a	3.5 b	5.5 a	1.2 a	2.1	6.2 b	6.8 b	0.6 a	3.0 b	3.8	6.8	2.6	7.4	8.1 a
Irrigation × Cultivar															
C_100_ × Cisco	4.4 c	4.8 c	6.2	3.8 c	0.5 b	2.0 b	8.0 b	8.4 a	nd	3.0 d	3.0	7.2	2.0	8.0	8.0 a
SDI_75_ × Cisco	5.0 b	5.4 b	4.0	5.0 b	0.2 c	1.5 c	8.8 a	9.1 a	nd	3.8 c	3.6	7.0	3.0	7.2	7.0 b
SDI_50_ × Cisco	7.6 a	5.4 b	5.5	5.0 b	1.2 b	2.4 b	8.8 a	8.8 a	nd	3.2 d	3.3	6.8	2.8	7.7	7.5 b
SDI_33_ × Cisco	3.8 d	2.2 e	2.6	3.2 c	1.0 b	2.5 b	6.9 c	7.0 b	nd	5.2 a	3.2	6.8	2.2	7.4	7.5 b
C_100_ × Chandler	4.0 c	7.2 a	6.6	5.4 a	0.7 b	1.3 c	6.4 d	6.8 b	1.5 a	2.8 d	3.4	6.9	2.0	7.8	8.2 a
SDI_75_ × Chandler	3.0 d	6.1 b	1.7	5.2 a	1.5 a	2.9 a	5.6 e	5.5 c	0.1 c	4.6 b	4.4	6.8	2.7	7.4	7.8 a
SDI_50_ × Chandler	3.0 d	6.2 b	3.8	5.6 a	1.2 b	1.5 c	6.0 d	6.8 b	0.2 c	2.4 e	3.6	6.8	2.7	7.2	8.2 a
SDI_33_ × Chandler	2.3 e	4.0 d	2.0	5.9 a	1.2 b	2.6 b	6.9 c	7.9 b	0.7 b	2.2 e	3.9	6.7	2.9	7.3	8.0 a

The scale ranged from 0 = no intensity to 10 = extremely strong intensity. SDI, sustained deficit irrigation; SDI_33_, at 33% ET_C_; SDI_50_, at 50% ET_C_; SDI_75_, at 75% ET_C_; and C_100_, control at 100% ET_C_. ^†^ NS = not significant; *, **, and *** = significant at *p* < 0.05, 0.01, and 0.001, respectively; and nd = not determined; ^‡^ Values (mean of 10 trained panelists) followed by different letters within the same column were significantly different (*p* < 0.05) according to Tukey’s least significant difference test.

**Table 8:** Sensory attributes, reference materials, and their corresponding intensities used for the descriptive analysis of walnuts.

Descriptor	Definition	Reference ^‡^	Intensity
	Appearance		
Color	The intensity of the color from light to dark	Figure 1	1.0−9.0
Size	The visual width of the almond from side to side	Figure 1	1.0−9.0
Veins	The visual vessels on the walnut skin	Figure 1	1.0−9.0
	**Basic Taste and Flavor**		
Sweetness	The basic taste associated with a sucrose solution	1% sucrose	5.0
		2% sucrose	7.0
Bitterness	The basic taste associated with a caffeine or quinine solution	0.01% caffeine	2.0
		0.02% caffeine	4.0
Astringency	A drying and puckering sensation on the mouth’s surface	0.05% alum	3.0
	**Flavor**		
Overall nuts	The aroma notes related to all nutty characteristics	Mix of ground Hacendado	7.0
Walnut ID	The aromatics associated with walnut	Ground Hacendado walnuts	8.0
Floral/Fruity	The sweet, light, and slightly perfumy impression associated with flowers and fruits such as apples and pears	Floral and fruity aroma of SOSA Ingredients^®^	8.0
Woody	The sweet, musty, dark, and dry aromatics associated with the tree bark	Ground Hacendado walnuts	3.0
Aftertaste	The longevity of key attributes after swallowing the sample	1 min	3.0
		2.5 min	5.0
		2.0 min	7.0
	**Texture**		
Hardness	The force needed to complete a bite through the sample with molar teeth, evaluated on the first bite down with the molars	Hacendado walnuts	8.0
		Hacendado almonds	10.0
Cohesiveness	The deformation degree of the sample prior to breaking when compressed between molars	Hacendado almonds	1.0
		Hacendado raisins	10.0
Crispiness	The intensity of audible noise perceived at first chew with molars	Biscuit “Galleta María” Hacendado	6.0
		Nestlé fitness	8.0
Adhesiveness	The work performed to completely eliminate the sample from the teeth	Hacendado almonds	3.0
		Hacendado raisins	10.0

^‡^ Intensities are based on a 10-point numerical scale with 0.5 increments, where 0 means “none” and 10 means “extremely strong”.

## Data Availability

The analyzed datasets are available from the corresponding author upon reasonable request.

## Abbreviations

The following abbreviations are used in this manuscript:

AA	Antioxidant activity
ET_C_	Crop evapotranspiration
FAMEs	Fatty acid methyl esters
MUFAs	Monounsaturated fatty acids
PUFAs	Polyunsaturated fatty acids
RDI	Regulated deficit irrigation
SDI	Sustained deficit irrigation
SFAs	Saturated fatty acids
TPC	Total phenolic content
WP	Water productivity
YR	Yield reduction
